# Differential human telomerase RNA knockdown by antisense oligonucleotides reveals the existence and potential function of a robust RNA domain

**DOI:** 10.1261/rna.081025.126

**Published:** 2026-08

**Authors:** Natalie Bao Ying Lim, Maya Jeitany, Peter Dröge, Kah Wai Lim, Anh Tuân Phan

**Affiliations:** 1 School of Physical and Mathematical Sciences, Nanyang Technological University, Singapore 637371, Singapore; 2 School of Biological Sciences, Nanyang Technological University, Singapore 637551, Singapore

**Keywords:** antisense oligonucleotides, robust RNA domain, telomerase RNA

## Abstract

Telomere length governs replicative capacity and cellular fate. In most cancers, telomeres are maintained by telomerase, a holoenzyme comprising an RNA component (hTR), a protein component (hTERT), and associated accessory proteins. hTR contains two major domains with distinct primary interactions: a 5′ domain that associates with hTERT, and a 3′ domain that binds core H/ACA proteins. Beyond telomere maintenance, hTR also exerts extra-telomeric roles. Here, we identified a potent antisense oligonucleotide (ASO) against hTR, which inhibited telomerase activity, leading to telomere shortening and cell growth impairment. Surprisingly, this ASO only mediated hTR degradation up to nucleotide 209, leaving the 3′ domain intact. Profiling of hTR using ASO tiling also supported this observation: whereas 3′-targeting ASOs could deplete the whole molecule, 5′-targeting ASOs could selectively deplete only the 5′ domain, leaving the 3′ domain intact, or even increase in abundance. Furthermore, the 3′ domain persisted at a steady state following prolonged treatment and was detected in untreated cells, suggesting its natural existence and potential function.

## INTRODUCTION

Telomeres, the repetitive DNA tracts capping chromosome ends (Blackburn 1991; Zakian 1995), partner with the shelterin complex (de Lange 2005) to protect these ends and preserve genome stability. Owing to the “end-replication problem,” telomeres progressively shorten every time the cell divides, limiting cellular proliferative capacity and ultimately triggering replicative senescence or apoptosis (Olovnikov 1973; Harley et al. 1990). Most cancer cells express telomerase, a specialized ribonucleoprotein (RNP) enzyme that counteracts telomere erosion by replenishing telomeric DNA that are lost in each cell division (Greider and Blackburn 1985; Morin 1989). Telomerase activity is coordinated by the shelterin and the CST complexes (Lim and Cech 2021). The core components of telomerase include telomerase reverse transcriptase (TERT) (Lingner et al. 1997; Weinrich et al. 1997), which catalyzes DNA synthesis, and telomerase RNA component (TR or TERC) (Greider and Blackburn 1989), which serves as the template for telomeric DNA synthesis as well as a scaffold for engaging protein partners.

The human telomerase RNA (hTR or hTERC), a 451 nucleotide (nt) molecule, exhibits a remarkably intricate architecture that integrates two major domains. The 5′ domain folds into a conserved template/pseudoknot (t/PK) domain critical for telomerase activity (Chen et al. 2000; Theimer et al. 2005). On the other hand, the 3′ domain adopts the H/ACA box structure (Kiss et al. 2010), composed of two tandem RNA hairpins connected by an H box and a ACA box. This 3′ domain recruits the canonical H/ACA box proteins (dyskerin, NHP2, NOP10, GAR1) and is essential for hTR stabilization and proper biogenesis (Mitchell et al. 1999; Nguyen et al. 2018). Additionally, hTR can form G-quadruplexes at its extreme 5′ end (Li et al. 2007), where such structures may protect the RNA from degradation during biogenesis (Sexton and Collins 2011). G-quadruplex formation can also occur within the P1 helix region, which impedes proper P1 helix assembly (Gros et al. 2008). Recent cryo-EM studies have confirmed the presence of a 5′ G-quadruplex within the telomerase holoenzyme and showed that it can coexist with P1 helix formation (Balch et al. 2025). Human telomerase has been proposed to be dimeric (Sauerwald et al. 2013), and in recent years, its dimeric structure has been resolved (Balch et al. 2025). This dimeric configuration, found in a small subset of telomerase, is critical for telomerase assembly and forms through interactions between hTR and H/ACA RNPs. Overall, these structural motifs support the role of hTR in coordinating telomerase activity, stability and assembly.

Given its essential role in telomere maintenance and elevated expression in most cancers (Soder et al. 1997), there has been keen interest in therapeutic targeting of hTR. Steric-blocking antisense 2′-O-methoxyethyl oligonucleotides (Chen et al. 2002), peptide nucleic acids (PNA) (Norton et al. 1996) and thio-phosphoramidates (Shea-Herbert et al. 2002), as well as degradation-based 2-5A conjugates (Kondo et al. 1998) and ribozymes (Yokoyama et al. 1998), showed good activity in vitro but were largely limited by poor cellular uptake (Duncan et al. 1998). To date, Imetelstat (GRN163L), a lipid-conjugated thio-phosphoramidate telomerase inhibitor, remains the sole clinically approved example (Lennox et al. 2024).

Significant advances in oligonucleotide chemistry have enabled the design of antisense agents with high potency and favorable therapeutic profile. Gapmer antisense oligonucleotides (ASOs), comprising a central DNA segment flanked by chemically modified nucleotides, represent an important innovation. Upon hybridization with target RNA, gapmer ASOs work by exploiting endogenous RNase H1 enzyme to cleave the targeted RNA (Crooke et al. 2021). Following ASO-mediated cleavage, both the 5′ and 3′ fragments can be subject to degradation by RNA surveillance machinery (Lima et al. 2016).

Beyond its canonical role with telomerase, hTR has also been implicated in extra-telomeric functions. Its expression in normal somatic cells (Yi et al. 1999), along with its stoichiometrically higher expression in telomerase-expressing cells (Xi and Cech 2014), suggest alternative roles. Indeed, hTR has been shown to regulate the proliferation and apoptosis in cancer cells and CD4^+^ T cells (Li et al. 2004; Gazzaniga and Blackburn 2014), modulate DNA damage responses (Kedde et al. 2006), mediate cellular processes via its association with chromatin (Chu et al. 2011; Liu et al. 2019; Wu et al. 2022) and with other RNAs (Ivanyi-Nagy et al. 2018), and even encode a functional protein (hTERP) involved in autophagy regulation (Rubtsova et al. 2018). The modular nature of hTR raises the possibility that its individual domains may engage in noncanonical roles.

In this study, we designed gapmer ASOs targeting hTR and identified a potent ASO candidate against the t/PK domain with low-nanomolar half-maximal inhibitory concentration (IC_50_) through free uptake, thereby overcoming barriers in cellular delivery. Depletion in hTR suppressed telomerase activity, shortened telomeres and eventually impaired clonogenic potential. Unexpectedly, this ASO treatment did not result in similar depletion of the 5′ and 3′ fragments, and instead induced degradation only of the 5′ domain, up to nucleotide 209, leaving behind a 3′ domain (residues 209–451), which overlapped with the H/ACA box domain. ASO tiling experiments reinforced this observation: while ASOs targeting the 3′ domain could destroy the whole hTR, ASOs targeting the 5′ domain could only deplete the 5′ domain, leaving the 3′ domain intact, or even increase in abundance. The elevated level of the 3′ domain was found more prominent with increasing dose of ASO; this level eventually converged back to the baseline level after ASO removal, indicating that hTR production is likely regulated by some form of compensatory mechanism. A steady-state level of the 3′ domain remained even after prolonged treatment, suggesting possibilities of its natural existence. Indeed, low levels of this isolated 3′ domain were detected endogenously in untreated cells, suggesting its natural presence. Depletion of the 3′ domain impaired clonogenic growth, though to varying extent, suggesting its potential function.

## RESULTS

### Identification of a potent gapmer ASO against hTR

**FIGURE 1.**
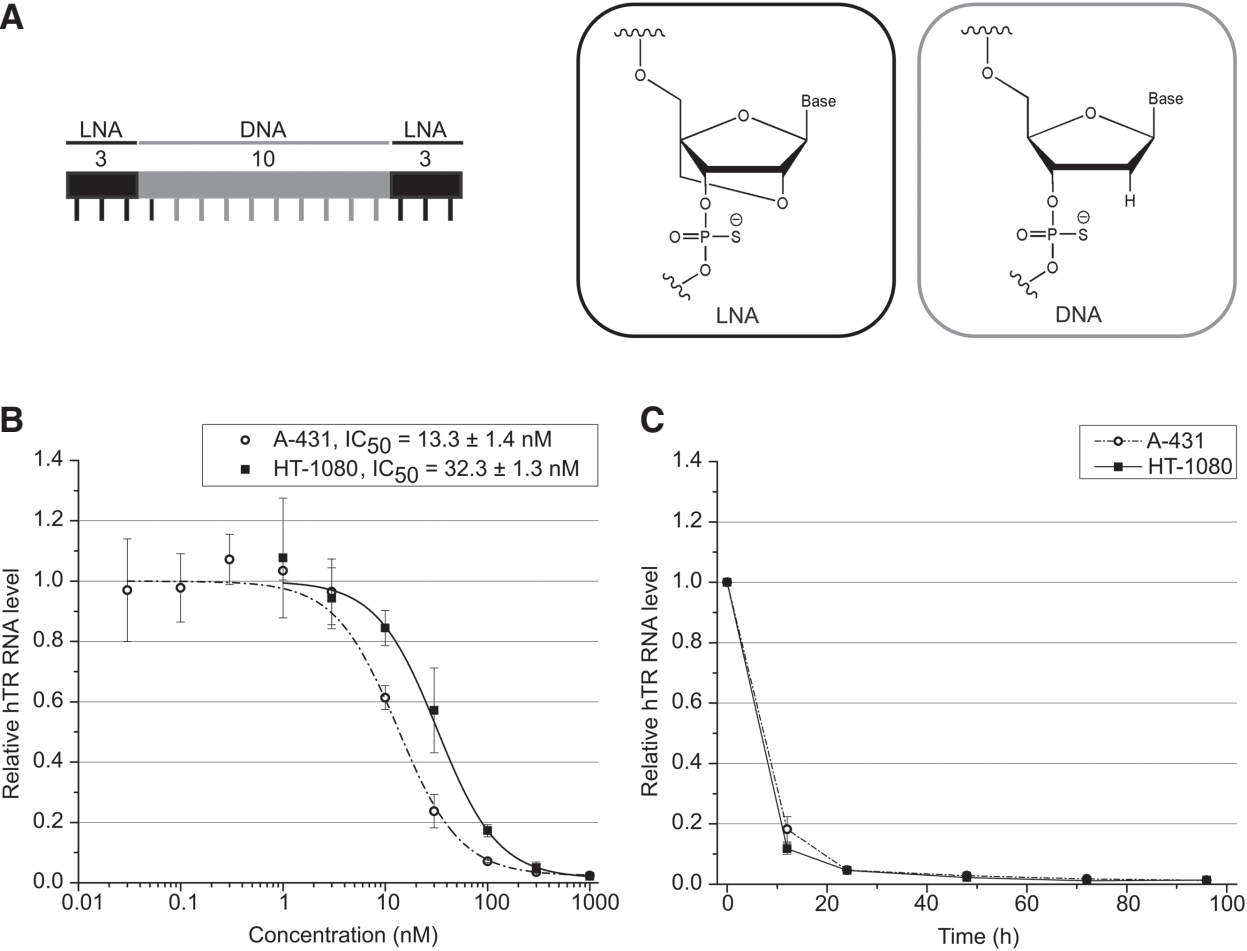
Gapmer ASO G042 exhibits potent knockdown activity against hTR. (*A*) Schematic of the gapmer ASOs used in this study, with a 3-10-3 (LNA-DNA-LNA) chemical configuration. (*B*) hTR RNA levels in A-431 and HT-1080 cells treated with varying doses of gapmer ASO G042 for 2 days. Values represent means (±SD) from three biological replicates, normalized to NTC-treated samples. (*C*) hTR RNA levels in A-431 and HT-1080 following treatment with 5 μM G042 for 12–96 h. Values represent means (±SD) from three biological replicates, normalized to NTC-treated samples.

To identify effective ASOs against hTR, we designed gapmer ASOs comprising full phosphorothioate (PS) linkages and locked nucleic acid (LNA) chemistry in a 3-10-3 LNA-DNA-LNA configuration (Fig. 1A; Monia et al. 1993). An initial screening was performed using real-time quantitative reverse transcription polymerase chain reaction (qRT-PCR) to measure hTR RNA levels in A-431 cells following treatment in vitro at a dose of 5 μM (Supplemental Table S1; Supplemental Fig. S1A–C). RNA levels were normalized to cells treated with a nontargeting control (NTC) gapmer comprising the same chemical modification pattern. From our preliminary screening, we identified two regions (nucleotides 25–51 and nucleotides 140–165) showing the highest hTR knockdown, with gapmer ASO G042 emerging as one of the most potent candidates. To assess target specificity, we designed two additional ASO controls (Gagnon and Corey 2019): a scramble (Scr) ASO consisting the same chemistry and nucleotide composition but randomized sequence, and a mismatch (MM) ASO containing three nucleotide substitutions relative to ASO G042 (Supplemental Table S1). G042 demonstrated a clear dose-dependent hTR RNA depletion with IC_50_ of 13.3 ± 1.4 nM in A-431. Similarly, G042 is also potent in HT-1080, with IC_50_ of 32.3 ± 1.3 nM (Fig. 1B). Time-course measurements revealed over 95% RNA reduction after 24 h, which was sustained up to at least 96 h (Fig. 1C). Notably, efficient hTR RNA reduction was achieved through free uptake (otherwise known as gymnosis [Crooke et al. 2021]), without the use of transfection reagents.

### G042 effectively inhibits telomerase activity, shortens telomere length and decreases cell viability over time in telomerase-positive A-431 and HT-1080 cells

**FIGURE 2.**
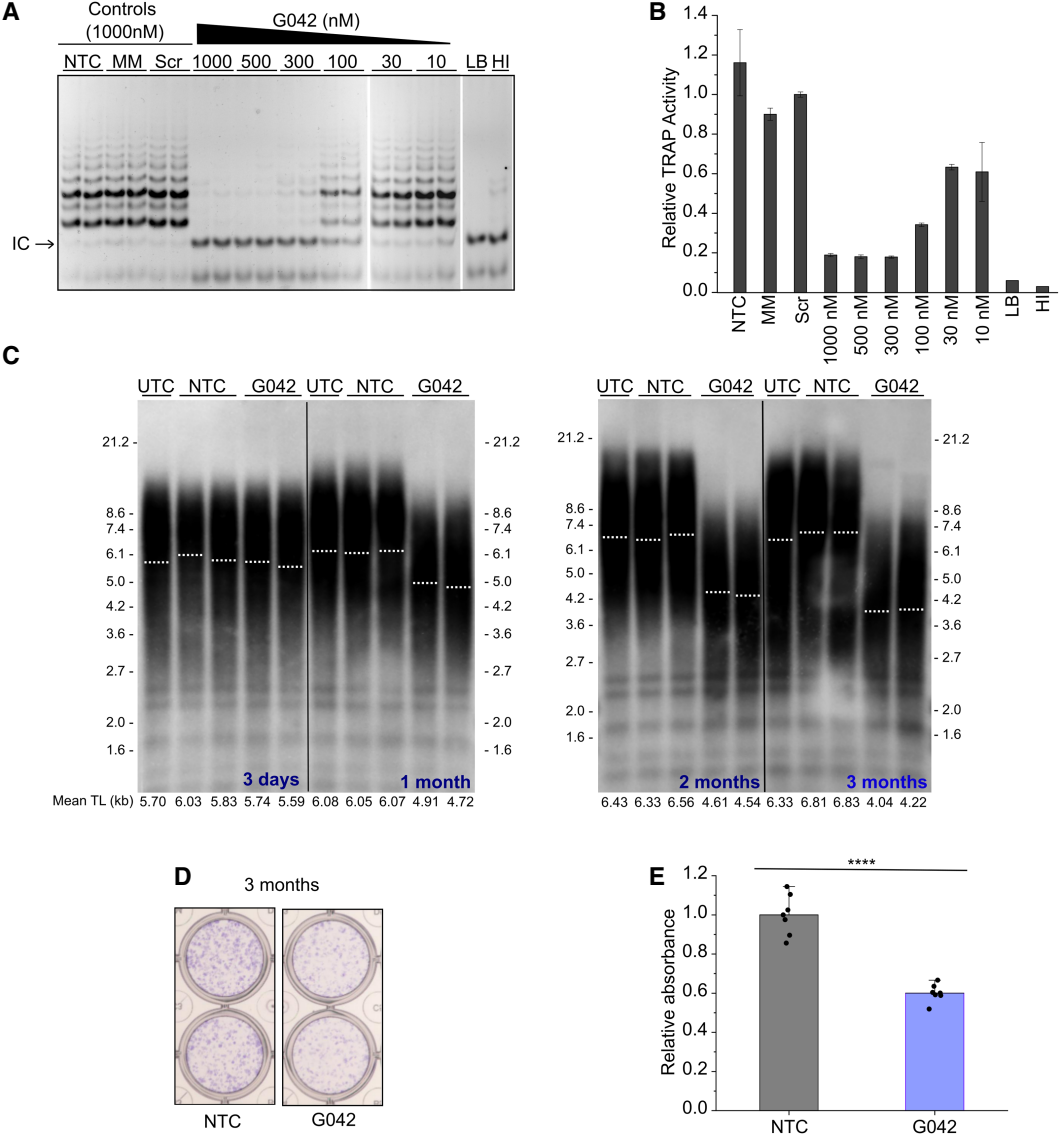
G042 effectively inhibits telomerase activity, shortens telomere length and decreases cell viability over time in HT-1080 cells. (*A*) Representative TRAP assay on HT-1080 cells following 72 hours of treatment with varying concentrations of ASO as indicated. Control ASOs were treated at 1000 nM. Lysis buffer (LB)-only and heat-inactivated (HI) samples were included as negative controls. IC represents the internal control product. (*B*) Quantification data of (*A*), values are from two biological replicates. (*C*) TRF analysis of HT-1080 cells treated with NTC ASO or G042, or untreated control (UTC), at indicated time points (3 days, 1, 2, and 3 months). (*D*) Clonogenic assays on HT-1080 following prolonged NTC ASO or G042 treatment for 3 months, with corresponding quantification of the relative colony area in *E*. Values are relative means (±SD) from two biological replicates. (****) *P*<0.0001, as determined by two-tailed unpaired *t*-test.

To examine the effect of hTR RNA suppression on telomerase function, we employed the telomerase repeat amplification protocol (TRAP) assay to measure telomerase activity of the ASO-treated cells. G042 inhibited telomerase activity in a concentration-dependent manner in both HT-1080 (Fig. 2A,B) and A-431 cells (Supplemental Fig. S2A,B), whereas the three control ASOs had no effect, confirming G042's on-target activity. Long-term telomerase inhibition is expected to progressively shorten telomere length to a critical threshold (∼1–2 kb), at which point cells undergo senescence or apoptosis (Martens et al. 2000). To assess this, telomere lengths of HT-1080 and A-431 were monitored using the telomere restriction fragment (TRF) assay. Cells were treated twice weekly with NTC or G042 at a dose of 500 nM and harvested at various time points for analysis. In HT-1080 cells, which possess inherently long telomeres, mean telomere lengths (TLs) decreased sharply from ∼6.1 to ∼4.8 kb during the first month, with further gradual shortening over the next 2 months. By the end of 3 months, mean TLs in G042-treated cells were effectively suppressed to ∼4.1 kb. The apparent slower rate of telomere shortening may be attributable to the concurrent telomere elongation, as observed for the untreated control (UTC) and NTC-treated control cells (Fig. 2C; Pickett et al. 2009) masking the true extent of telomere shortening. After correcting this, telomere shortening per month is estimated to be ∼0.6–1 kb per month. This spontaneous telomere elongation of the control cells in HT-1080 is consistent with previous reports of its natural telomere elongation (Pickett et al. 2009). A-431 cells treated with G042 showed less pronounced TL shortening (Supplemental Fig. S2C), with the caveat that TRF analysis was limited to 1 month due to low cell viability. The less pronounced signal in A-431 cells is likely due to the loss of cells with the shortest telomeres to senescence or death as well as the inherent difficulty of visualizing sub-2 kb telomeres by TRF due to hybridization kinetics (Lai et al. 2018). Functionally, long-term treatment reduced clonogenic capacity, manifesting after 3 months in HT-1080 (Fig. 2D,E), and as early as 2 months in A-431 (Supplemental Fig. S2D,E). Collectively, these results demonstrate G042 as a specific and potent inhibitor of telomerase, leading to telomere shortening and diminished clonogenic potential.

### ASOs targeting the 5′ region leave the 3′ region of hTR intact

**FIGURE 3.**
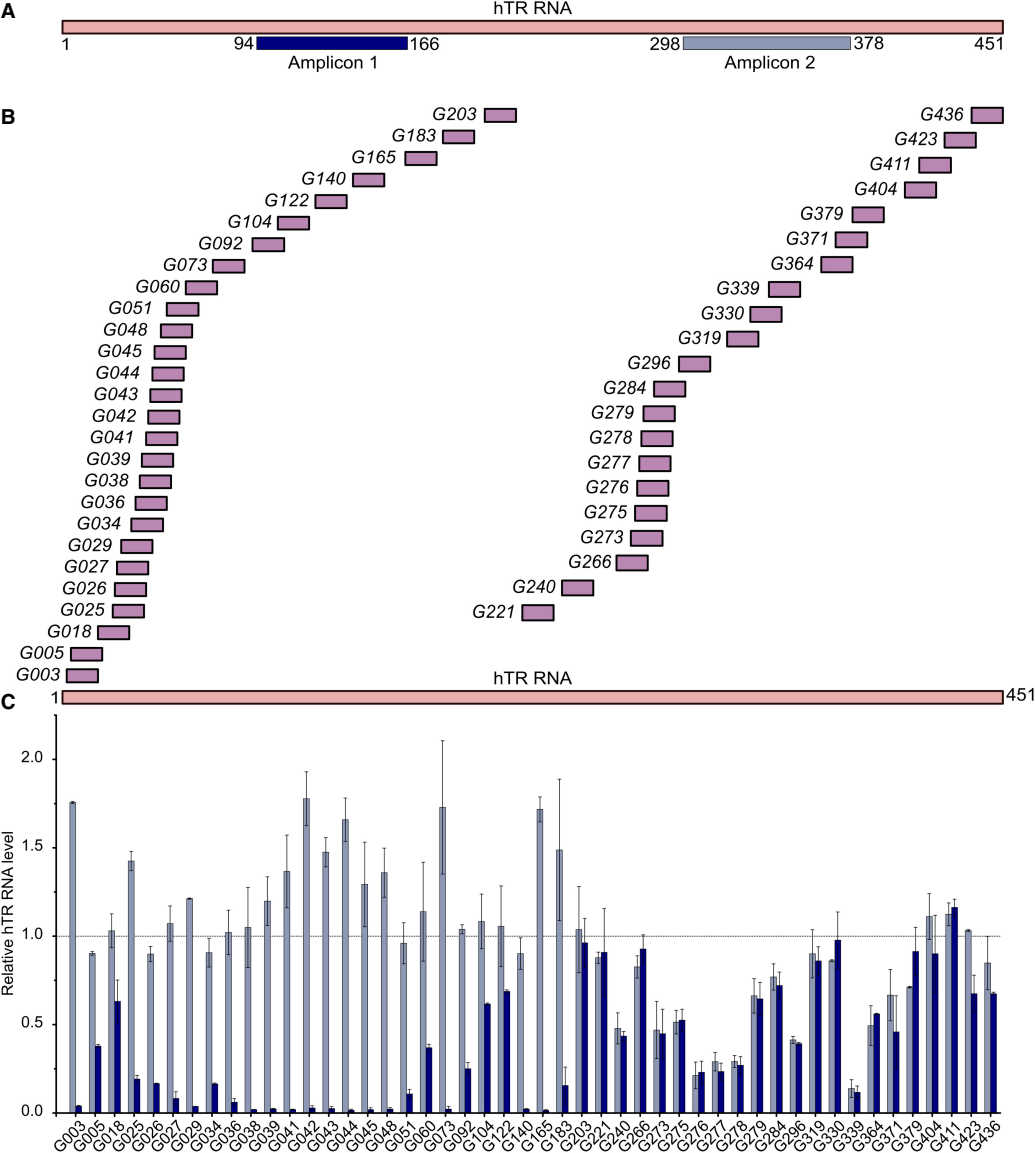
ASOs targeting the 5′ region leaves the 3′ region of hTR intact. (*A*) Schematic showing the positions of the two hTR amplicons, amplicon 1 (spanning 5′ region of hTR) and amplicon 2 (spanning 3′ region of hTR). (*B*) Schematic showing the targeting sites of the panel of 48 gapmer ASO candidates. (*C*) hTR RNA levels in A-431 cells treated with the 48 gapmer ASOs following a treatment of 2 days at 5 μM. hTR RNA levels were measured by real-time quantitative PCR (qRT-PCR) based on the two amplicons. Values are from at least two biological replicates.

Up to this point, we observed potent knockdown of hTR with G042 based on an amplicon spanning nucleotides 94–166 (amplicon 1). Following ASO-mediated cleavage, both upstream and downstream fragments can be further degraded by RNA surveillance machinery (Lima et al. 2016). Unexpectedly, no knockdown was observed when we switched the detection to an amplicon spanning nucleotides 298–378 (amplicon 2) (Fig. 3A; Supplemental Fig. S3A). Systematically probing hTR levels across the same panel of 48 gapmer ASOs (Fig. 3B; Supplemental Table S1) using the second amplicon revealed a striking, distinctive hTR degradation pattern: while ASOs targeting the 5′ region of hTR (left side) showed a significant mismatch in readout between the two amplicons, ASOs targeting the 3′ region of hTR (right side) showed similar readouts from both amplicons (Fig. 3C). This pattern indicates that the active 5'-targeting ASOs strongly depleted amplicon 1 (5′ domain), but did not reduce, and in some cases even elevated, the level of amplicon 2 (3′ domain). In contrast, the depletion by 3′-targeting ASOs is uniform, showing consistent measurements of the two amplicons. These findings not only corroborated our initial observation with G042, a 5′-targeting ASO, but also uncovered an interesting phenomenon: whereas 3′-targeting ASOs trigger uniform degradation, 5′-targeting ASOs, if active, selectively degrade only the 5′ domain, leaving with the 3′ domain mostly intact. To further characterize the fate of this truncated fragment, we assessed whether ASO treatment altered its subcellular localization. We performed nuclear-cytoplasmic fractionation and measured the level of hTR with the two amplicons following G042 treatment. hTR was detected in both the nuclear and cytoplasmic compartments. In the UTC sample, the results on amplicon 1 indicated that the 5′-domain-containing species (likely mainly the full-length hTR) was present predominantly in the nucleus, while with amplicon 2 we only detected a slightly higher abundance of the 3′ domain in the nucleus as compared to cytoplasm. In the treated sample, the 5′ domain was depleted across all fractions, while the 3′ domain showed no significant changes in its levels across fractions and its subcellular localization (Supplemental Fig. S3B,C).

### Dynamic interplay between the two regions of hTR reveals a potential compensatory feedback mechanism

**FIGURE 4.**
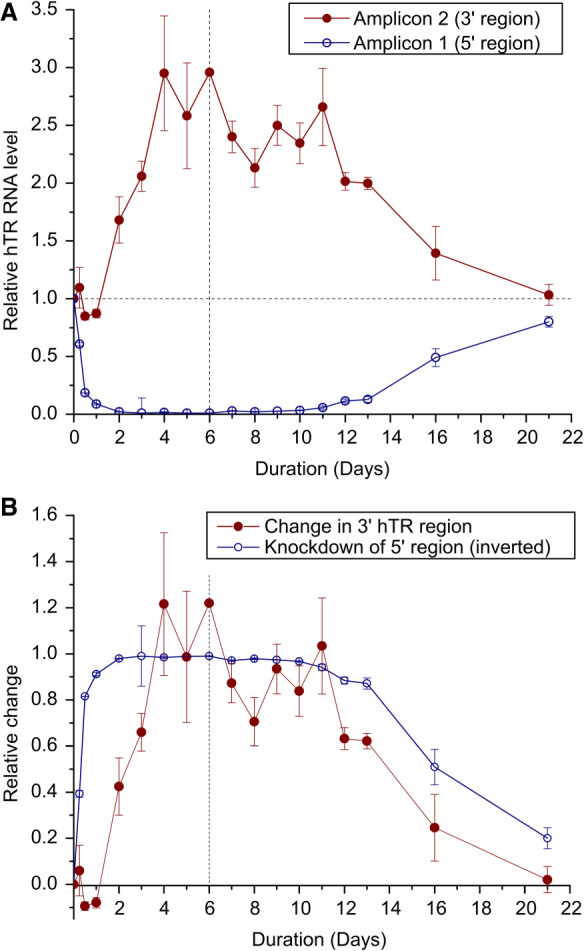
Dynamic interplay between the two regions of hTR reveals a potential compensatory feedback mechanism. (*A*) hTR RNA levels in A-431 cells were measured by qRT-PCR using the two amplicons. Cells were treated with gapmer G042 at 5 μM for 6 days, after which the ASO was removed from cell culture medium. Levels were normalized to NTC-treated cells, which were similarly treated for 6 days (at 5 μM) before ASO removal. Values are from at least two biological replicates. (*B*) Replot of (*A*) to observe the time-lag relationship between the two regions. The amplicon 1 curve was inverted by plotting *y* = −*y*, where *y* = relative hTR RNA level. The amplicon 2 curve was transformed to represent normalized relative change in 3′ hTR region using (y-1)/(<y_d4-d8_>-1), where <y_d4-d8_> represents the average RNA levels from days 4 to 8.

An intriguing inverse correlation between the two hTR domains was observed: increased degradation of the 5′ region (amplicon 1) was accompanied by a concomitant increased abundance of the residual 3′ region (amplicon 2) (Fig. 3C), particularly for ASOs G003, G029, G039-G048, G073, and G165. To further examine this relationship, A-431 cells were treated with G042 for 6 days, followed by ASO removal, and the hTR RNA levels were monitored via both amplicons throughout the experiment. G042 treatment at 5 μM resulted in a sharp decrease in the 5′ region of hTR, while the 3′ region exhibited a delayed but robust increase in abundance, peaking at approximately threefold by day 4 and sustained through day 6 (Fig. 4A). Following ASO removal at day 6, the 5′ region gradually increased, returning to its baseline expression. At the same time, the 3′ region followed this trend, ultimately converging to the baseline level. Treatment with varying doses also supported this trend, which showed higher ASO concentrations producing stronger initial upregulation of the 3′ region of hTR (amplicon 2, in agreement with Fig. 3C), followed by a gradual return to the baseline expression after ASO withdrawal (Supplemental Fig. S4). To visualize the time-lag relationship, data from Figure 4A were replotted by transforming the curves to represent the 5′ region knockdown and the normalized relative change in the 3′ region. This revealed a time-lag of ∼2 days for the 3′ region to be upregulated in response to the depletion of the 5′ region (Fig. 4B). Collectively, our time- and dose-dependence data reveal an inverse correlation between the two regions of hTR, pointing to some form of compensatory feedback mechanism that is activated when the 5′ region of hTR is perturbed.

### Mapping the 5′ boundary of the residual hTR RNA domain using 5′ RACE technique

**FIGURE 5.**
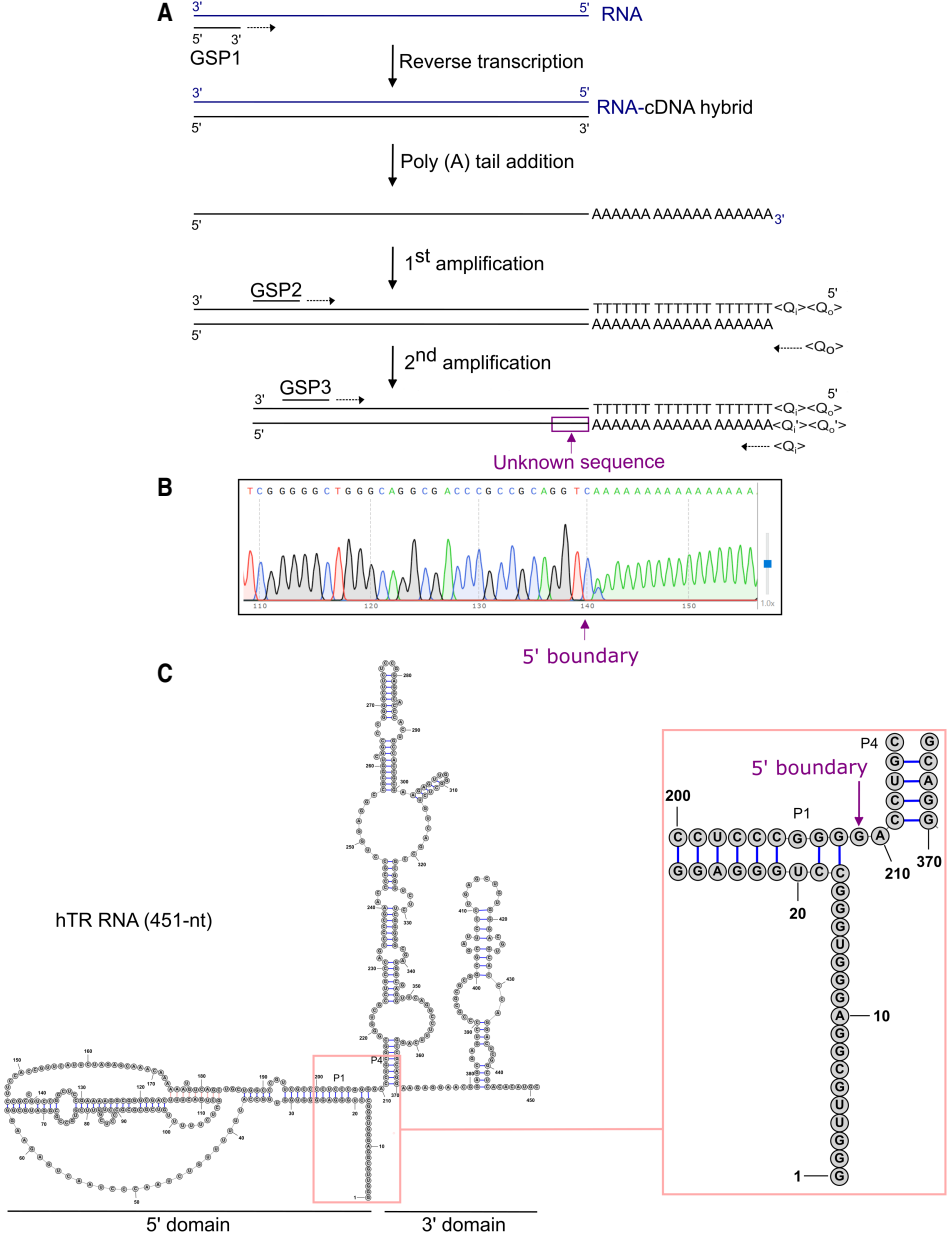
Mapping the 5′ boundary of the residual hTR RNA domain using 5′ RACE technique. (*A*) Simplified schematic representation of the classic 5′ RACE technique. Reverse transcription with a gene-specific primer (GSP) produces cDNA. The RNA strand is degraded by RNase H, releasing the cDNA strand. Terminal transferase then adds a poly(A) tail to the cDNA, providing a priming site for an oligo(dT) primer in PCR together with another GSP. Finally, nested PCR with additional primers increases specificity, allowing reliable detection of unknown 5′ ends. (*B*) Sanger sequencing chromatogram showing the sequence of the 5′ RACE product of G042-treated sample in A-431 cells. Purple arrow indicates the approximate 5′ boundary of the residual RNA domain. (*C*) Schematic representation of the hTR secondary structure, along with the approximately mapped 5′ boundary at the junction connecting the two domains.

We next sought to define the 5′ boundary of the residual hTR RNA fragment. To map the 5'-end of the remaining RNA domain, we employed the classic 5′ rapid amplification of cDNA ends (5′ RACE) assay (Fig. 5A). Sanger sequencing of the 5′ RACE product, followed by BLAST alignment next to the primer-derived poly(A) tail, placed the 5′ boundary of the residual fragment at approximately nucleotide 209 (Fig. 5B; Supplemental Fig. S5). This location lies at the P1–P4 junction connecting the two domains (Fig. 5C). The precise 5′ boundary is subjected to few nucleotides variation due to the intrinsic terminal transferase activity of the reverse transcriptase used, which might incorporate two to four additional cytosines (Scotto-Lavino et al. 2006). In summary, this site corresponds to where ASO-induced hTR cleavage stops, marking the start of the stable 3′ domain, a region overlapping with the canonical H/ACA box structure of hTR.

### The 3′ domain of hTR is maintained at steady-state level within cells after prolonged ASO treatment

**FIGURE 6.**
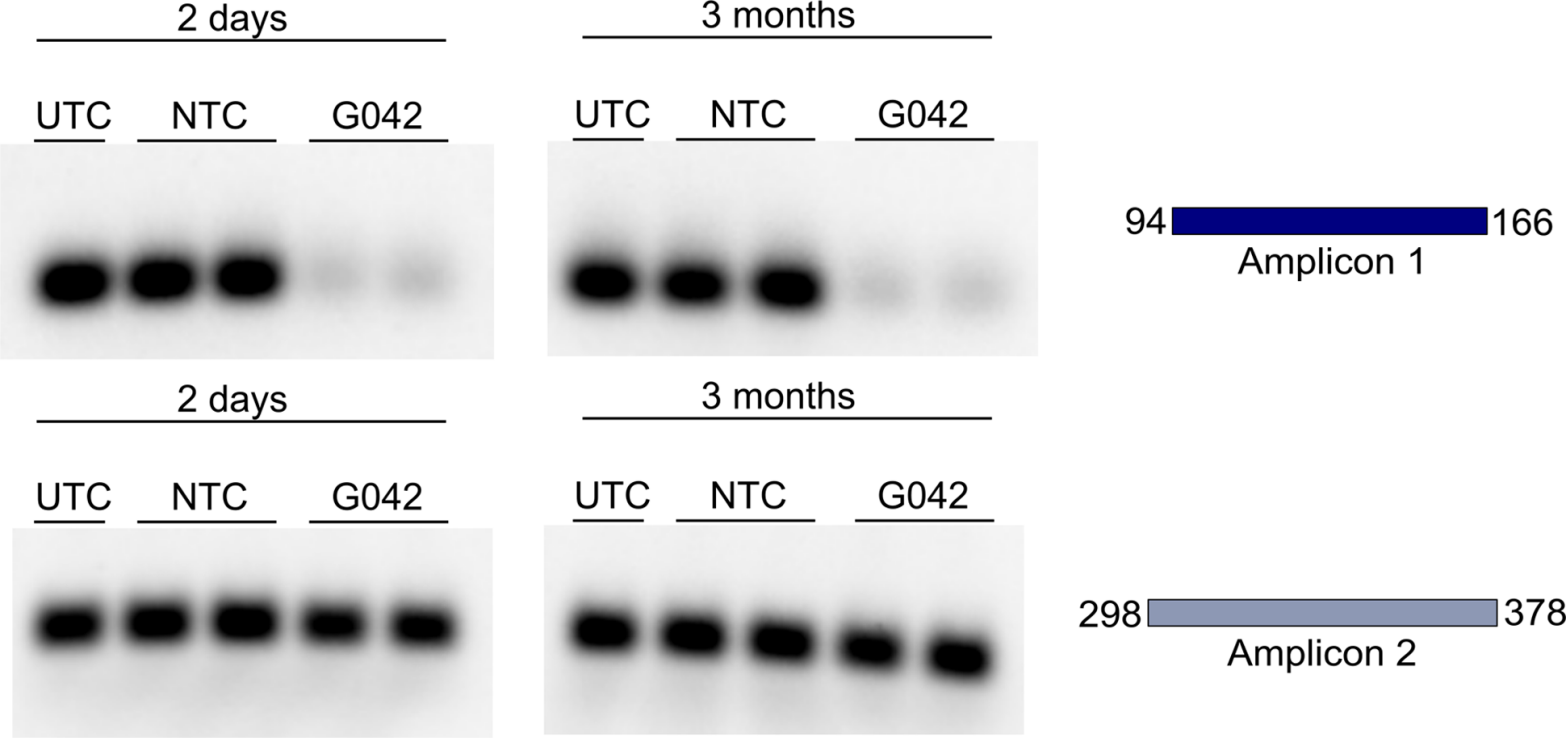
The 3′ domain of hTR is maintained at steady-state level within cells after prolonged ASO treatment. PCR bands from HT-1080 cells treated with 500 nM of NTC ASO or G042 for 2 days (*left*) and 3 months (*right*). Detection was done using primers spanning amplicons 1 (*top*) and 2 (*bottom*). UTC samples were included as an additional control. Twenty-eight cycles of PCR amplification were performed.

To examine if the 3′ domain of hTR generated by 5′-targeting ASO treatment remains detectable after prolonged period, we analyzed HT-1080 cells after 3 months of continuous G042 (5′-targeting ASO) treatment (long time point). Our results showed the same trend as observed with short-term treatment (Fig. 6; Supplemental Fig. S3A), with the 5′ domain depleted while the 3′ domain remaining readily detectable in G042-treated samples (Fig. 6; Supplemental Fig. S6A,B). These results indicate that the truncated 3′ fragment is not rapidly eliminated by cellular RNA surveillance mechanism but instead persists at a steady-state level with continuous ASO treatment. Importantly, no additional mechanisms appear to be activated to eliminate this fragment, suggesting that a pool of the truncated 3′ hTR domain is tolerated in cells.

### The truncated 3′ hTR domain may exist in the natural context

**FIGURE 7.**
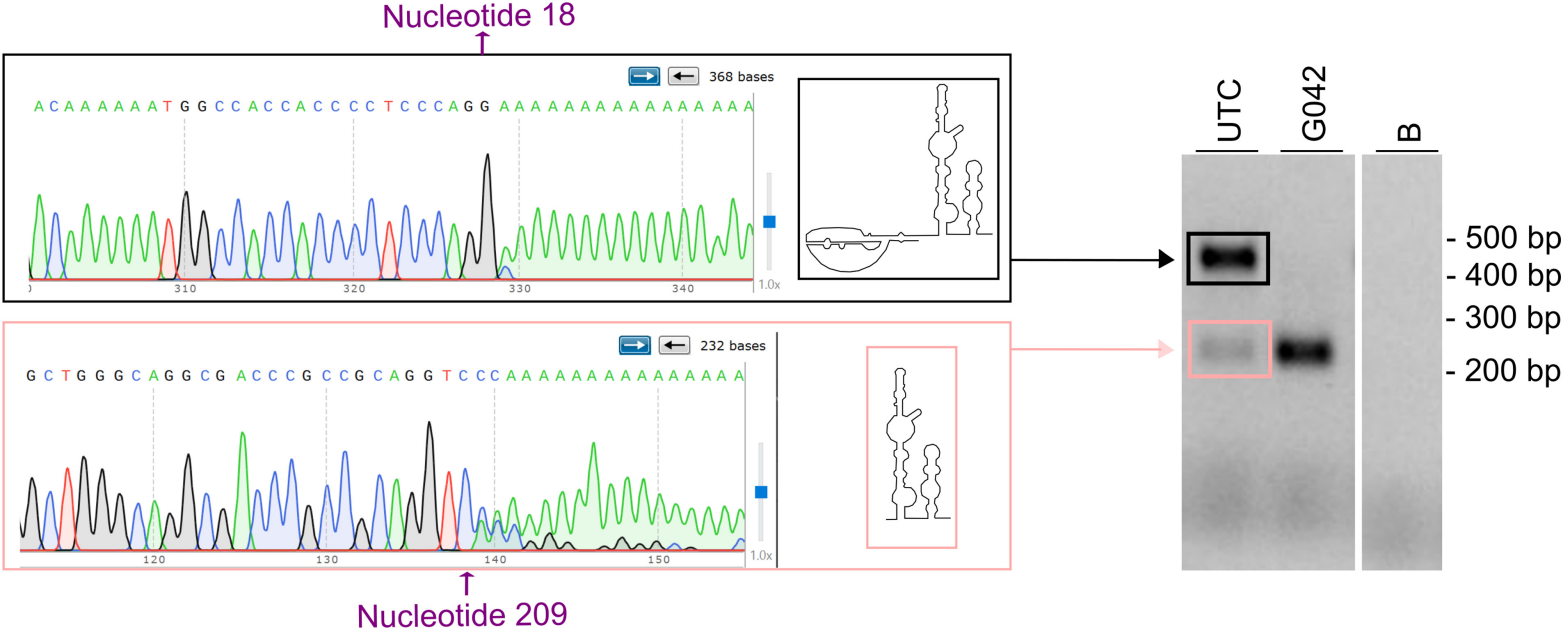
The truncated 3′ hTR domain may exist in the natural context. Gel analysis of the 5′ RACE products of UTC versus G042-treated samples, along with a blank (B, water-only) control. Two 5′ RACE products are present in the UTC sample. Sanger sequencing chromatogram showing the sequence of the top 5′ RACE product (framed in black) and the bottom 5′ RACE product (framed in pink). Purple arrow indicates the approximate 5′ boundary of each product.

The steady-state population of the 3′ hTR domain under long-term ASO exposure raises intriguing questions about its natural existence in cells. To investigate this, we performed the 5′ RACE assay on the UTC sample of A-431 cells, which yielded two distinct 5′ RACE products: a larger band above 400 bp and a smaller band corresponding in size to the residual fragment observed in G042-treated cells (Fig. 7). Sanger sequencing of the top band shows that it is mapped near nucleotide 18, corresponding closely to the full-length hTR product (Supplemental Fig. S7A), rather than nucleotide 1. The 5′ terminus (nucleotide 1–18) is capable of forming a stable G-quadruplex (Li et al. 2007), a structure that is also recently resolved in cryo-EM analyses (Balch et al. 2025). There are two possibilities that can account for this observation: either the 5′ terminus cannot be reverse transcribed due to G-quadruplex impediment, or that the 5′ terminus itself is truncated. Nonetheless, Sanger sequencing of the bottom band (Fig. 7; Supplemental Fig. S7B) revealed an exact match to the previously characterized 3′ hTR domain after ASO treatment, again mapping the 5′ end near nucleotide 209. These findings suggest that the residual 3′ hTR domain that we had previously observed with ASO-treated cells may be present naturally, albeit at low levels.

### The abundance of the 3′ hTR domain varies across cell lines

**FIGURE 8.**
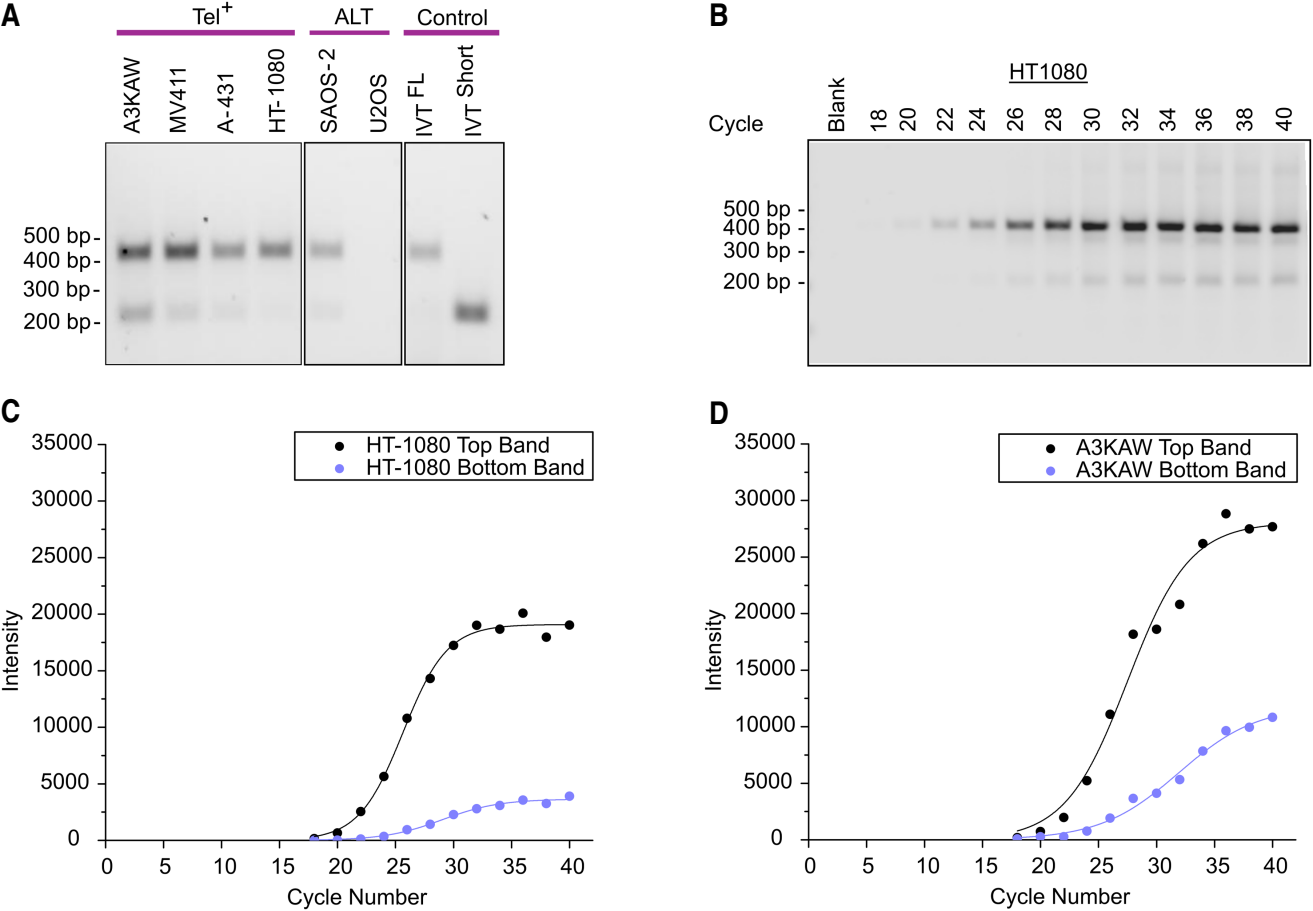
The abundance of the 3′ hTR domain varies across cell lines. (*A*) Gel analysis of the 5′ RACE products from some representative Tel^+^ and ALT cell lines, along with IVT hTR as controls. Twenty-six cycles of PCR amplification were performed. (*B*) The gel of the 5′ RACE experiment showing changes in the intensity of top and bottom bands in HT-1080 with increasing number of cycles. (*C*) Quantification of the top and bottom bands in HT-1080 across the cycle numbers. (*D*) Quantification of the top and bottom bands in A3KAW across the cycle numbers. Raw gel is shown in Supplemental Figure S8A.

To investigate how the relative proportion of the full-length hTR and the short fragment (3′ domain) vary among different cell lines, we performed the same 5′ RACE technique on UTC samples from a few telomerase-positive (Tel^+^), along with SAOS-2, an alternative lengthening of telomeres (ALT)-positive cells. As experimental controls, we concurrently included the in vitro transcribed (IVT) full-length hTR (IVT^FL^) and the short hTR isoform (IVT^Short^), alongside the ALT-positive U2OS cell line, which was reported not to express hTR (Akıncılar et al. 2015). With the exception of U2OS, all tested cell lines, regardless of their status (Tel^+^ or ALT), were found to contain this short hTR fragment, albeit at varying abundance (Fig. 8A). This fragment was also present in normal fibroblast cells IMR90 (Supplemental Fig. S8A), suggesting that this is not a unique feature of immortal cell lines. Among the tested cell lines, the abundance of the short fragment was found to be the highest in A3KAW cells and the lowest in HT-1080. To quantitatively monitor the relative abundance of the two hTR population, we performed PCR with varying numbers of amplification cycles and monitored the two populations by gel electrophoresis. The intensities of the two bands were then plotted as qPCR amplification curves for HT-1080 (Fig. 8B,C) and A3KAW cells (Fig. 8D; Supplemental Fig. S8B). The differences in quantification cycle (ΔCq) between the top and bottom bands ranged from ∼4.1–4.5 in A3KAW and ∼4.9–5.2 in HT-1080, corresponding to ∼4.2%–5.5% and ∼2.6%–3.2% of the short fragment in A3KAW and HT-1080, respectively (Supplemental Fig. S8C,D). While it remains unclear why some cells contain a higher proportion of the short species, it is generally present across all tested cell types (except for hTR-negative U2OS cells).

### The impact of the 3′ hTR domain depletion on A-431 and HT-1080 cells

**FIGURE 9.**
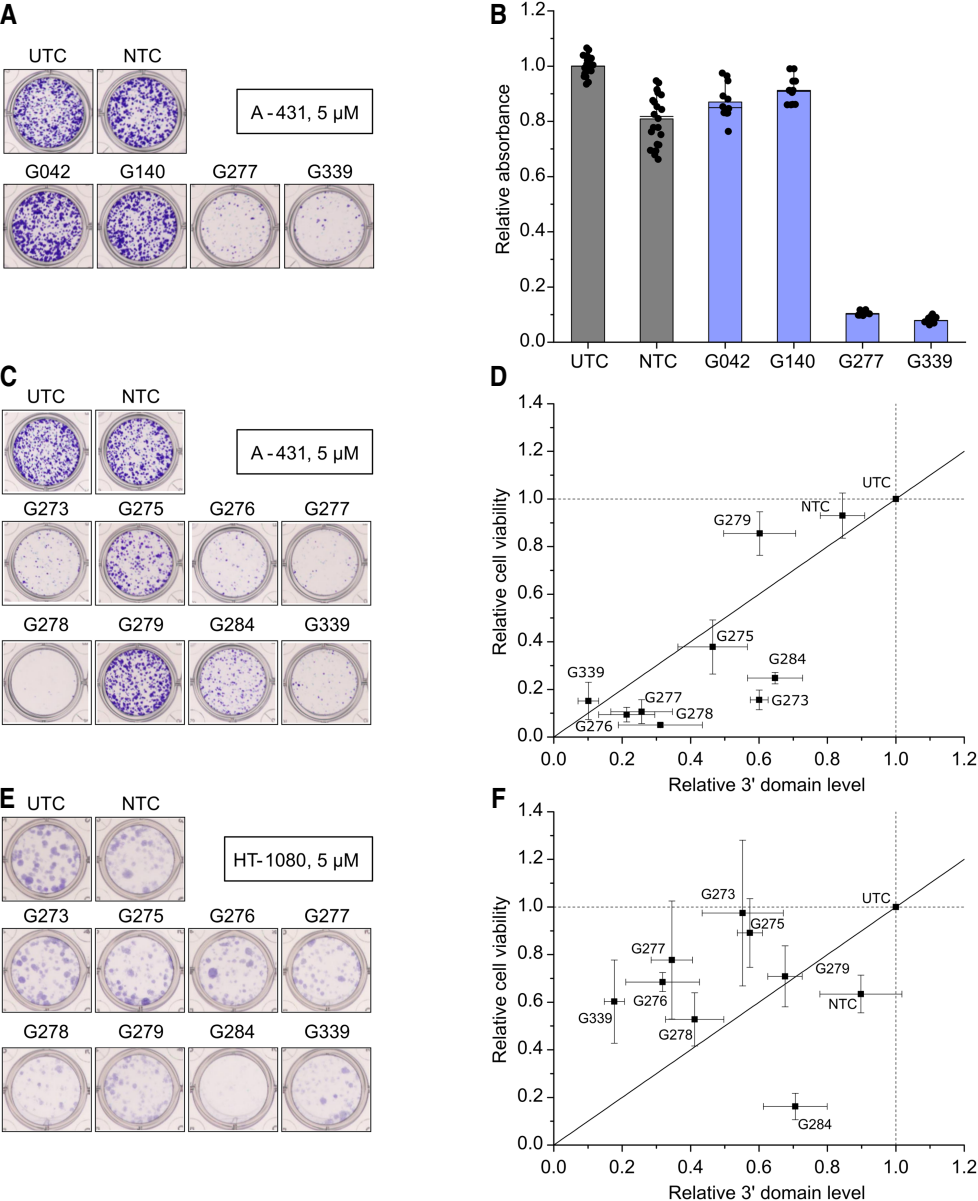
The impact of the 3′ hTR domain depletion on A-431 and HT-1080 cells. (*A*) Colony formation assays on A-431 following 5 μM treatment with NTC, partial hTR-degrading ASOs (G042, G140) and full-length hTR-degrading ASOs (G277, G339). Duration of this treatment is short-term (9 days). Quantification of the absorbance of stained and lyzed colonies is indicated in *B*. (*C*) Colony formation assays on A-431 following 5 μM treatment with NTC ASO and a series of ASOs degrading both domains of hTR. Duration of treatment is short-term (9 days). (*D*) Correlation plot showing the relationship between the relative cell viability and the 3′ hTR domain level in *C*. The line *y* = *x* was drawn to visualize the correlation. Relative cell viability was measured by examining the relative absorbance values from each well. The 3′ domain level was measured with amplicon 2, which was also consistent with the measurement by amplicon 1. Values are relative mean (±SD) from at least three biological replicates. (*E*) Colony formation assays on HT-1080 following 5 μM treatment with NTC ASO and a series of ASOs degrading both domains of hTR. (*F*) Correlation plot showing the relationship between the relative cell viability and the 3′ hTR domain level in *E*. The line *y* = *x* was drawn to visualize the correlation. Relative cell viability was measured by examining the relative absorbance values from each well. The 3′ domain level was measured with amplicon 2, which was also consistent with the measurement by amplicon 1.

To assess the functional impact of the 3′ hTR domain depletion, we treated A-431 cells for 9 days with ASOs that deplete both domains and ASOs that only deplete the 5′ domain. We then examined the cell survival potential at both 5 μM (Fig. 9A,B) and 2.5 μM (Supplemental Fig. S9A,B). Clonogenic assays showed impaired colony formation in cells treated with ASOs that reduce the 3′ domain/both domains (G277, G339), whereas ASOs that degraded only the 5′ domain (G042, G140) showed minimal effect on its clonogenic potential at this time scale. To further assess the dependency of this 3′ domain for survival, we treated the cells sequentially: G042 was first added to deplete the 5′ domain (and enrich the 3′ domain population). Then, either NTC, ASOs depleting the 3′ domain/both domains (G278, G339) or ASOs depleting only the 5′ domain (G042, G140) were administered to evaluate the effect of depleting the 3′ domain on colony formation. Only cells treated with ASOs targeting the 3′ domain/both domains exhibited impaired clonogenic growth, whereas cells treated with NTC or ASOs depleting only the 5′ domain were unaffected (Supplemental Fig. S9C,D), further supporting a role the 3′ domain may have in sustaining cell survival. Next, we treated different cell lines with a series of ASOs that deplete the 3′ domain/both domains to visualize the correlation between the level of 3′ hTR domain and colony formation. In A-431 cells, a good correlation was observed between the knockdown of 3′ hTR domain and the reduction in clonogenic potential (Fig. 9C,D). A correlation was also observed in HT-1080, but to a lesser extent (Fig. 9E,F). These results show that the 3′ hTR domain may have some roles in cell survival, but the dependency on this domain for survival varies across cells.

## DISCUSSION

Human telomerase RNA (hTR or hTERC) has long been perceived as an attractive therapeutic target due to its essential role in telomere maintenance. However, hTR's significance extends beyond this canonical function. Normal somatic cells also express hTR (Yi et al. 1999). In telomerase-positive cells, a substantial fraction of hTR is not bound to hTERT (Xi and Cech 2014). These observations strongly suggest extra-telomeric functions for hTR.

In this study, we employed gapmer ASOs targeting hTR, achieving robust depletion of hTR with low-nanomolar potency through free cellular uptake. Treatment with gapmer G042 inhibited telomerase activity, shortened telomeres and ultimately reduced cell survival potential. We observed a more rapid decrease in colony-forming ability in A-431 than in HT-1080 cells, which aligns with the expected vulnerability of cells with short telomeres (A-431) to telomerase inhibition. These phenotypic outcomes are consistent with the well-established canonical role of hTR with telomerase.

The differential efficiencies observed across our ASO panel (Supplemental Table S1) may be explained by the region-specific accessibility of hTR during RNP assembly. Following transcription, nascent hTR first associates with pre-H/ACA complex (dyskerin, NOP10, NHP2, NAF1) (Qin and Autexier 2021), with recruitment of hTERT to the t/PK and CR4/5 domains occurring at a later stage (Egan and Collins 2012). Consistent with this order of events, we observed highest ASO efficiencies for the t/PK region, particularly at the single-stranded template sequence, which remains accessible in both mature TERT-bound and TERT-free hTR. Conversely, ASOs targeting the 3′ domain generally showed lower efficiencies, likely because of the limited accessibility in both free and TERT-bound forms.

Importantly, our ASO tiling screening revealed striking, site-dependent distinctions in hTR degradation pattern, highlighting variable domain resistance to nuclease cleavage. While 3′-targeting ASOs induced hTR depletion uniformly with the two amplicons, a clear disparity was observed for the 5′-targeting ASOs. More specifically, the 5′-targeting ASOs, if active, depleted the 5′ domain, but left the 3′ domain intact, or even increased in abundance (Fig. 3C). These findings are consistent with earlier reports that ASO-mediated cleavage does not always result in the uniform depletion of an entire molecule (Hasselblatt et al. 2005). We further show here that the degradation outcome depends on both the specific ASO targeting site and the structural features of the target RNA. Subcellular analysis shows that a small pool of hTR exists in the cytoplasm, consistent with previous reports (Sun et al. 2024; Zhang et al. 2025). Upon targeting the 5′ domain, the residual 3′ hTR fragment is still found in both the nucleus and cytoplasm.

Interestingly, we observed an inverted correlation in which the depletion of the 5′ region correlated with enhancement of the residual 3′ region (Figs. 3C, 4A; Supplemental Fig. S4). Upon depletion of the 5′ region, cells may sense the loss and compensate by enhancing hTR RNA production, resulting in the accumulation of the 3′ region, which reached up to threefold increase at the highest ASO dose tested. Following the removal of ASO and recovery of the 5′ region, a balance is restored between the two regions. How this sensing mechanism operates, however, warrants further investigation.

Using the 5′ RACE technique, we mapped the truncated 3′ domain following G042 treatment to residues 209–451, a region overlapping with the H/ACA box domain (Fig. 5B,C; Supplemental Fig. S5). Although the hTR H/ACA domain has previously been detected as a stable form in transfected cells expressing recombinant hTR using northern blot analysis (Mitchell et al. 1999), it was not endogenously detected as an independent domain in wild-type cells.

Using a sensitive PCR-based method, we are now able to detect the 3′ domain (∼2%–5%) across the tested cell lines endogenously under native cellular conditions (Fig. 8A), supporting its potential physiological relevance. This endogenous fragment may arise through a processing pathway similar to that observed for recombinant hTR molecules. Its stability is likely supported by extensive protein (e.g., H/ACA box proteins) and RNA interactions within this region (Ivanyi-Nagy et al. 2018; Nguyen et al. 2018), such as the interactions between hTR and histone mRNAs (Ivanyi-Nagy et al. 2018). The RNA–RNA interaction at this region, together with compartmental sequestration, may aid resistance to ASO-mediated degradation. While the interactions of the 3′ hTR domain with these protein and RNA partners were not experimentally confirmed in this study, this represents an important direction for future work.

Our findings suggest that the 3′ domain may be important in sustaining cell viability: while ASOs that deplete only the 5′ domain did not affect its viability, we observed a good correlation between knockdown of the 3′ domain of hTR and clonogenic potential in A-431 cells within a short time scale of 9 days (Fig. 9C,D). These effects are unlikely to be a result of telomere shortening, a process we have previously shown to take months to manifest (Supplemental Fig. S2D,E), suggesting a pathway independent of telomerase inhibition.

The potential role of the H/ACA domain in promoting cell survival observed here is consistent with findings by Gazzaniga and Blackburn, who reported that telomerase-inactive hTR mutants can protect stimulated CD4^+^ T cells from apoptosis. Importantly, replacing the H/ACA domain of hTR with the H/ACA domain of the similarly sized U64 small nucleolar RNA abolished this protective effect, indicating that the function is specific to the native 3′ hTR domain (Gazzaniga and Blackburn 2014). Its role in sustaining clonogenic growth may come from telomerase-independent interactions within the H/ACA domain of hTR with other mRNAs, such as its interaction with key apoptosis regulators (Ivanyi-Nagy et al. 2018). Depleting the 3′ domain of hTR may affect the interactions with these key apoptosis regulators and affect cellular viability. However, this depletion does not impact the viability of A-431 and HT-1080 cells equally.

Structurally, hTR can adopt diverse conformations beyond the canonical form (Chen et al. 2000; Theimer et al. 2005). These structures include a pseudoknot-lacking conformer within the t/PK domain and alternate folding within the CR4/5 domains (Forino et al. 2025). Apart from its full-length form, shorter variants of hTR have been reported, such as TERC-53 (Cheng et al. 2018), and 3′-end-derived small RNAs like T3p (Fish et al. 2018) and *terc*-sRNA (Laudadio et al. 2019). The primers in this study are not expected to amplify these species. The independent 3′ domain detected here represents another hTR form and broadens the known molecular repertoire of hTR.

Nonetheless, technical limitations should be acknowledged. RNA secondary structures (e.g., G-quadruplexes) and the terminal transferase activity of reverse transcriptase may affect the mapping of the 5′ RACE products by a few nucleotides. This does not change our finding that the truncated domain overlaps with the H/ACA domain. Some effects on cell viability by the ASOs targeting 3′ domain/both domains may also arise from off-target interactions that cannot be fully predicted algorithmically. Nevertheless, a correlation was observed between multiple ASOs and cell viability, suggesting a dependence on the H/ACA domain for cell survival.

Overall, our findings highlight variable domain resistance of hTR to nuclease degradation event and show the existence of the 3′ hTR domain as an independent domain. This region also encompasses the CR4/5 domain, which normally serves as a binding interface for TERT (Nguyen et al. 2018), and contains the P4 stem implicated in TERT dimerization (Balch et al. 2025). Alternative CR4/5 conformations have been observed (Forino et al. 2025), and whether depleting the 5′ domain changes the proportion of hTR adopting these alternative conformations remains unknown. Moreover, selective depletion of the 5′ domain removes the t/PK contact point between hTR and hTERT, potentially exposing that region of hTERT, enhancing interactions with other protein or RNA partners that may modulate or enhance its extra-telomeric functions.

Collectively, our results reinforce hTR's intricate role as a multifunctional lncRNA and establish gapmer ASOs as functional probes in RNA biology, offering both therapeutic and fundamental value. ASOs have previously been employed as probes of RNA structures. These include ASOs that disrupt RNA-RNA/RNA-protein interactions for studying ribonucleoprotein complex assembly (Sheng et al. 2025), and ASOs that mediate RNase H cleavage to improve RNA secondary structure prediction (Kauffmann et al. 2009). Here, we extend the RNase H-based approach and show that the two different domain-specific readouts can provide complementary structural information for the characterization of a multi-domain RNA structure. We anticipate that this strategy can also be generalized to study the structures of other RNAs.

## MATERIALS AND METHODS

### Cell culture

Human fibrosarcoma HT-1080 cells, human epidermoid carcinoma A-431 cells (CRL-1555), human acute monocytic leukemia MV411 and human osteosarcoma U2OS cells were purchased from ATCC while human lymphoma A3KAW cells were purchased from JCRB Cell Bank. Human osteosarcoma SAOS-2 cells and normal fibroblast IMR90 cells were received from Professor Dennis Kappei at National University of Singapore (original source: ATCC). A-431, SAOS-2, HT-1080, and U2OS cells were grown in Dulbecco's modified Eagle's medium (DMEM, Gibco). MV411 and A3KAW cells were cultured in Roswell Park Memorial Institute medium 1640 (RPMI, Gibco). All culture media were supplemented with 10% Fetal Bovine Serum (FBS, Gibco) and 1× Penicillin/Streptomycin (Gibco) at 37°C with 5% CO_2_. Cultures were routinely checked for mycoplasma.

### Design and synthesis of gapmer ASOs

All 16 nt ASOs comprising a 3-10-3 gapmer configuration with fully phosphorothioate backbone (Monia et al. 1993) were designed across the full length of hTR RNA (RefSeq identifier NR_001566.3). Synthesis of all ASOs was done with an in-house DNA/RNA synthesizer (ABI 394). Owing to occasional technical issues with our synthesizer, some ASO batches were purchased from IDT. All ASOs obtained from IDT were tested against their in-house counterparts, confirming comparable activities. In-house synthesis employed standard solid phase phosphoramidite chemistry and commercially available reagents sourced from Glen Research (Controlled pore glass [CPG] supports, DNA/LNA phosphoramidites) and ChemGenes Corporation (Phenylacetyl disulfide as the sulfurizing agent). Post synthesis, the ASOs underwent cleavage and deprotection following manufacturer's instructions. Subsequently, the ASOs were purified by either reverse phase HPLC (RP-HPLC) or Glen-Pak DNA Purification Cartridge (Glen Research), followed by desalting using Glen Pak 2.5 desalting column (Glen Research). After lyophilization, the ASOs were dissolved in Phosphate Buffered Saline (PBS) (Gibco) and reconstituted to 100 μM stock solutions before use. The JEOL SpiralTOF MALDI-TOF mass spectrometer was used for the characterization of all ASOs.

### Gapmer ASO treatment

For the treatment of gapmer ASOs, cells were first seeded on 24 well plate (Corning) a day before treatment. To attain the desired dosing, ASOs were diluted with fresh culture media and introduced directly to each treatment well, without using any additional carriers or transfection reagents.

### Real-time quantitative reverse transcription polymerase chain reaction (qRT-PCR)

HT-1080 and A-431 cells were plated at 30,000 and 80,000 cells per well respectively on 24 well plates (corning) 1 day before ASO treatment. Cells were then harvested 48 h post-gapmer treatment, and total RNA was extracted from each culture well using either TRIzol reagent (Thermo Fisher Scientific) for low-throughput experiments or the MGIEasy Total RNA Extraction Kit (MGI 940-000875-00) for high-throughput applications, following the manufacturer's guidelines. The MGIEasy kit includes on-bead DNase treatment, whereas TRIzol-extracted RNA was subjected to RQ1 RNase-Free DNase (Promega) treatment prior to cDNA synthesis. cDNA was synthesized using the M-MLV reverse transcriptase (Promega) kit with gene specific primers (GAPDH: AAGTGGTCGTTGAGGGCAATG and hTR: ATGTGTGAGCCGAGTCCTG). Following reverse transcription, Thermolabile Exonuclease I (NEB) was used to remove residual single-stranded primers. The resulting cDNA was diluted 1:10 before being subjected to qRT-PCR using iTaq Universal SYBR Green Supermix (Bio-Rad) on a Bio-Rad CFX96 real-time PCR detection system. Relative target expression level was determined with the 2^−ΔΔ^ threshold cycle method, using GAPDH as a reference (Livak and Schmittgen 2001). The primer pairs used are shown in Table 1.

**TABLE 1.** List of qRT-PCR primers

Gene	Forward primer (5′ → 3′)	Reverse primer (5′ → 3′)
GAPDH	CTGGGCTACACTGAGCACC	AAGTGGTCGTTGAGGGCAATG
hTR_Amplicon 1 (nt 94–166)	CGCTGTTTTTCTCGCTGACT	GCTCTAGAATGAACGGTGGAA
hTR_Amplicon 2 (nt 298–378)	GCGAAGAGTTGGGCTCTGTCA	TTCCTCTTCCTGCGGCCTGAAA

### Telomeric repeat amplification protocol (TRAP) assay

Cells were plated and treated following the same protocol used for qRT-PCR. Cells were subsequently harvested 72 h after ASO treatment. A nonradioactive modification of the TRAP assay was adapted in this study, following the methodology described by Herbert et al. (2006). Briefly, reaction mixture containing 2 µg of protein extract in NP-40 lysis buffer and 100 ng of Cy5-TS oligo were incubated at 20°C for 60 min, followed by a heating step at 95°C for 5 min to inactivate telomerase. Subsequently, the DNA amplification reaction was prepared by adding the reverse primer ACX, internal standard control primer TSNT, reverse primer NT for internal standard, and Taq DNA Polymerase (Thermo Scientific). Touchdown PCR cycle was then performed using these cycling conditions: initial denaturation at 95°C for 5 min; three cycles of 95°C for 30 sec, 60°C for 30 sec and 72°C for 45 sec; three cycles of 95°C for 30 sec, 55°C for 30 sec and 72°C for 45 sec; 29 cycles of 95°C for 30 sec, 52°C for 30 sec and 72°C for 45 sec; and a final extension at 72°C for 10 min. The internal standard normalizes the signals for differences in PCR efficiency. The reaction mixture was run on a 10% native polyacrylamide gel in 0.5× TBE (14.5 mA constant current, 5.5 h), and scanned using the Amersham Imager 680 (Cytiva). Lanes containing heat-inactivated (HI) cell extract- and lysis buffer (LB) only- samples were used as negative controls for the assay. The oligonucleotides used in this assay are shown in Table 2.

**TABLE 2.** List of oligonucleotides used in the TRAP assay

Oligonucleotide	Sequence (5′ → 3′)
Cy5-TS oligo	Cy5-AATCCGTCGAGCAGAGTT
Reverse primer ACX	GCGCGGCTTACCCTTACCCTTACCCTAACC
Internal standard control primer TSNT	AATCCGTCGAGCAGAGTTAAAAGGCCGAGAAGCGAT
Reverse primer NT	ATCGCTTCTCGGCCTTTT

### Telomere restriction fragment (TRF) assay

Genomic DNA was isolated from cells using the DNeasy Blood & Tissue Kit (Qiagen). Briefly, 1–2 μg of gDNA was subjected to restriction digestion with HinfI and RsaI enzymes (New England Biolabs) for 6 h at 37°C and run on 0.8% agarose gel at 37 V for 16 h. Digestion quality was evaluated by gel staining using 0.5 µg/mL ethidium bromide (Bio-Rad) in water. Then, the gel was destained in water, followed by washes with HCl solution, denaturation solution and neutralization solution, according to the protocol of TeloTAGGG Telomere Length Assay Kit (Roche). DNA was then capillary transferred overnight to a Hybond-N + nylon membrane (GE Healthcare) using 20× sodium citrate buffer. UV crosslinking twice at 120 mJ using HL-2000 Hybrilinker (UVP Laboratory Products) was then performed the following day. The rest of the hybridization and antibody incubation steps were done following manufacturer's protocol. Mean telomere length estimation was performed using the WALTER webtool (Lyčka et al. 2021).

### Clonogenic assays

One day before gapmer treatment, ∼200 HT-1080 cells or 1500 A-431 cells were plated per well in 24 well plates. After incubation for 7–9 days, methanol was added to fix the cells, following which colony staining solution (0.5% [w/v] crystal violet [Sigma-Aldrich] in 25% methanol) was applied. Subsequently, the plates were rinsed with water to remove excess stain and left to dry overnight at ambient temperature.

### Classic 5′ RACE

The classic 5′ RACE was performed as reported by Scotto-Lavino et al. (2006). Total RNA was first reverse transcribed with hTR gene-specific reverse transcription primer (QSP1) using M-MLV reverse transcriptase (Promega), following the manufacturer's protocol. The obtained first-strand cDNA product was appended with a poly(A) tail using terminal deoxynucleotidyl transferase (Thermo Scientific) at 37°C for 5 min followed by a 10 min deactivation step at 70°C, before it was subjected to the first round PCR amplification using the hybrid primer Q_T_, Q_o_ primer, and GSP2. All PCR amplifications were done using Q5 Hot Start High-Fidelity DNA Polymerase (NEB). For the first round of PCR, the following program was used: 98°C for 40 sec, 49°C for 15 sec, 72°C for 30 sec for one cycle, followed by 27 cycles of 98°C for 10 sec, 55°C for 10 sec, and 72°C for 30 sec; and a final extension at 72°C for 2 min. A second round of PCR was then performed using nested primers (Q_I_ and GSP3) to improve the formation of specific PCR products. The following program was used: initial denaturation at 98°C for 30 sec, followed by 26 cycles of 98°C for 10 sec, 60°C for 10 sec, 72°C for 30 sec and a final extension at 72°C. The obtained amplicons were run on a 2%-agarose gel stained with SYBR Safe (Thermo Scientific), and the products were gel-extracted for purification (GeneJET PCR Purification Kit, Thermo Scientific) and sent for Sanger sequencing. The primers used are shown in Table 3.

**TABLE 3.** List of primers used in the 5′RACE assay

Primer	Sequence (5′ → 3′)
GSP1	ATGTGTGAGCCGAGTCCTG
GSP2	GACTCGCTCCGTTCCTCTTC
GSP3	TTCCTCTTCCTGCGGCCTGAAA
Q_T_	CCAGTGAGCAGAGTGACGAGGACTCGAGCTCAAGCTTTTTTTTTT TTTTTTT
Q_O_	CCAGTGAGCAGAGTGACG
Q_I_	GAGGACTCGAGCTCAAGC

GSP1-3 are gene-specific primers. Q_T_ is the tailing primer comprising the Q_O_ and Q_I_ primer-binding sites with a 1-nt overlap, followed by an oligo-dT stretch to anneal to the appended poly(A) tail.

### Preparation of standard IVT^FL^ and IVT^Short^

The template for in vitro transcription (IVT) of hTR was produced via a two-step PCR amplification strategy. In the first step, PCR products were generated using primers spanning full-length hTR (FL) and the 3′ hTR domain (short). Subsequently, a second PCR was performed using an elongated forward primer composed of the T7 promoter sequence and the same reverse primer. IVT was subsequently performed on the purified T7-hTR template using T7 RNA polymerase (NEB) following the manufacturer's instructions. Following transcription, the RNase-free DNase I (Promega) was used to remove the DNA template, and the transcribed hTR RNA was purified using phenol/chloroform extraction.

### Subcellular fractionation

Whole-cell, cytoplasmic and nuclear fractions were isolated using the PARIS kit (Thermo Fisher Scientific) following the manufacturer's instructions. The quality of fractionation was verified by western blot analysis of GAPDH (cytoplasmic marker) and Histone H3 (nuclear marker) and by RT-PCR of MALAT1 as a nuclear RNA marker.

### Western blot

Aliquots of the whole-cell, nuclear and cytoplasmic protein fractions were quantified using the BCA Protein Assay Kit (Merck Millipore). Approximately 8 µg of proteins were added in Laemmli sample buffer and denatured at 95°C for 10 min before loading into a 10% SDS-PAGE gel, after which electrophoresis was done at 100 V for 100 min. Following semi-dry transfer using the Trans-Blot Turbo System (Bio-Rad) onto PVDF membrane (GE Healthcare), the membrane was blocked using EveryBlot Blocking Buffer (Bio-Rad) for a duration of 5 min at ambient temperature and washed three times 5 min each with TBST before incubation with respective primary antibodies (GAPDH: 2118S; Histone H3: 9715S; Cell Signaling Technology) for 1 h at room temperature. After three more TBST washes, the membrane was incubated with HRP-labeled secondary antibodies (Anti-Rabbit IgG, HRP-linked Antibody: 7074S; Cell Signaling Technology) at room temperature for 1 h, followed by three final TBST washes. Finally, the membrane was incubated with ECL substrate (Bio-Rad), following the manufacturer's instructions, and imaged on Amersham Imager 680 (GE Healthcare).

### Statistical analysis

Statistical analyses were conducted as indicated in the respective figure legends. Data are shown as means (±SD) and represent measurements from minimally two independent experiments, unless stated otherwise.

## DATA DEPOSITION

All data are available in the main text or the supplemental material.

## SUPPLEMENTAL MATERIAL

Supplemental material is available for this article.

## COMPETING INTEREST STATEMENT

P.D. is a cofounder and shareholder of LamdaGen Pte. Ltd.

## Figures and Tables

**Natalie Bao Ying Lim:**
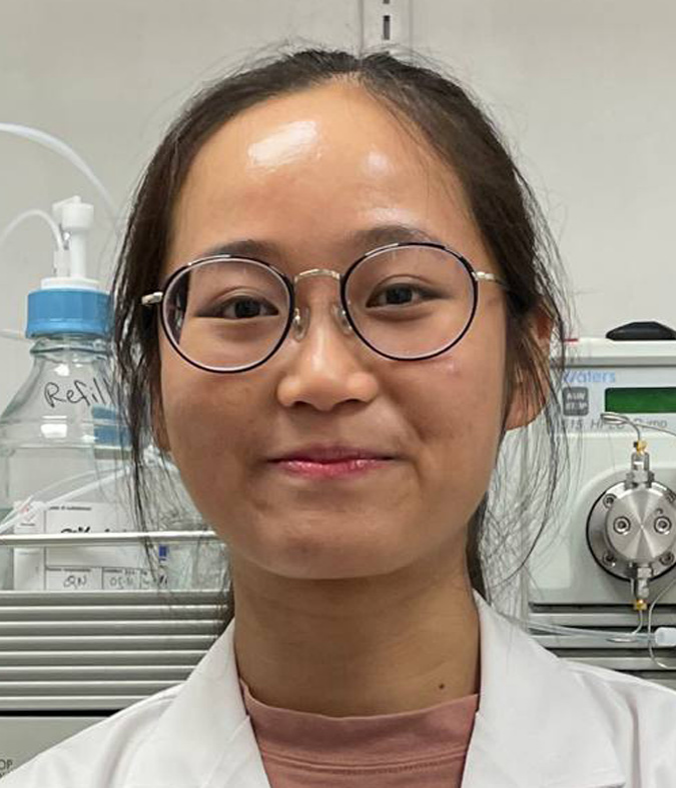


## MEET THE FIRST AUTHOR

***Meet the First Author(s)* is an editorial feature within *RNA*, in which the first author(s) of research-based papers in each issue have the opportunity to introduce themselves and their work to readers of *RNA* and the RNA research community. Natalie Bao Ying Lim is the first author of this paper, “Differential human telomerase RNA knockdown by antisense oligonucleotides reveals the existence and potential function of a robust RNA domain.” Natalie is a graduate student in the laboratory of Professor Anh Tuân Phan at Nanyang Technological University. Her research focuses on developing nucleic acid-based tools in telomere biology, using them as both therapeutic agents for cancer intervention and molecular probes for biological discovery.**

**What are the major results described in your paper and how do they impact this branch of the field?**

We demonstrated that the 3′ hTR domain stays intact, or even elevated, following treatment with ASOs targeting the 5′ domain. We further showed that the 3′ domain exists independently in native cellular condition, and that its depletion impacts cell viability, implicating a functional role. These findings reveal the existence of an additional form of hTR, adding a new dimension to hTR biology beyond its well-known role as the RNA component of the telomerase complex.

**What led you to study RNA or this aspect of RNA science?**

I was initially interested in the work of telomerase, an enzyme that could immortalize cells. Its RNA component, hTR, intrigued me because of how strikingly divergent this RNA is across species (despite the conserved function of the holoenzyme). In humans, telomerase RNA has co-opted the H/ACA box scaffold (a motif involved in pseudouridylation) for stability during biogenesis. What then surprised us was that this domain is resistant toward 5′-targeting ASOs. This prompted us to investigate where the ASO-mediated degradation halts, whether this domain exists naturally and if depleting this domain affects cellular viability.

**What are some of the landmark moments that provoked your interest in science or your development as a scientist?**

One landmark moment for me was discovering that we can actually make synthetic nucleic acids in the lab and engineer them to exploit endogenous cellular machinery across various applications (ASOs, siRNA, CRISPR guides, aptamers and more). It is remarkable to look at how far and fast the field has developed: from the fundamental discovery of various cellular machineries that process and recognize nucleic acids, to the development of chemical modifications that improve the pharmacological properties of these synthetic molecules while preserving their ability to engage their intended targets. But perhaps what I find most fascinating is how a four-letter code can fold into such a diverse repertoire of shapes and forms that can engage so many different cellular machineries.

**Are there specific individuals or groups who have influenced your philosophy or approach to science?**

I feel fortunate to be surrounded by amazing mentors throughout my PhD journey. My PhD supervisor, Professor Anh Tuân Phan, was incredibly supportive and genuinely nurturing. I am also grateful to the people in the biophysics lab, in particular Dr. Lim Kah Wai, as well as collaborators in the School of Biological Sciences, Professor Peter Dröge and Dr. Maya Jeitany, for all the guidance and mentorship throughout these few years. The opportunity to work on a project with scientists from such varied backgrounds was something that I valued, particularly in how it taught me to approach scientific problems through an interdisciplinary lens.
